# Intermittent upper limb cyanosis revealing Paget–Schroetter syndrome: the value of patient-provided photographs

**DOI:** 10.1186/s12245-026-01282-2

**Published:** 2026-07-02

**Authors:** Marine Desjardins, Giulia Casutt, Marco Fresa, Jean Perentes, Adriano Alatri, Pierre-Nicolas Carron

**Affiliations:** 1 Emergency Department, Lausanne University Hospital, Rue du Bugnon 44, Lausanne, CH-1011 Switzerland; 2 Division of Angiology, Lausanne University Hospital, Chemin du Mont-Paisible 18, Lausanne, CH-1011 Switzerland; 3 Division of Thoracic Surgery, Lausanne University Hospital, Rue du Bugnon 46, Lausanne, CH-1011 Switzerland; 4 Emergency Department, Lausanne University Hospital and University of Lausanne, Rue du Bugnon 44, Lausanne, CH-1011 Switzerland

**Keywords:** Paget–Schroetter syndrome, Venous thoracic outlet syndrome, Emergency department

## Abstract

**Background:**

Paget-Schroetter Syndrome (PSS) is a rare condition referring to primary upper extremity deep vein thrombosis occurring in the context of venous thoracic outlet syndrome (vTOS) The diagnosis is rarely considered in the emergency department (ED) and is difficult to establish when symptoms are intermittent or absent at presentation.

**Case presentation:**

A healthy 20-year-old woman presented three times in the ED, describing exertional right arm swelling and cyanosis. During consultations, she was slighty symptomatic. Duplex ultrasound showed axillary–subclavian thrombosis and computerized tomography scan (CT) phlebography revealed bilateral dynamic vTOS. Anticoagulation and thrombectomy were performed, with planned surgical decompression. The diagnosis was triggered by patient-provided photographs capturing intermittent symptoms.

**Conclusion:**

PSS may be missed in mild to moderate symptomatic ED presentations. Early vascular imaging and careful history-taking, including patient photographs, can enable timely treatment and prevent complications.

## Background

Paget-Schroetter Syndrome (PSS) is a rare condition referring to primary upper extremity deep vein thrombosis occurring in the context of venous thoracic outlet syndrome (vTOS) [1].

## Case presentation

A healthy 20-year-old woman presented to the ED for the third time, describing intermittent cyanosis and swelling of the right upper extremity, occurring after repeated forward flexion movement of the arm and accompanied by mild subaxillary pain and transient neurological signs (mild paresis and paresthesia of the right-hand fingers). Symptoms resolved within minutes when the arm was extended. At the time of examination patient refer just a feeling of heaviness of right arm without any other symptoms.

She reported no trauma, cervical or thoracic pain, cardiopulmonary symptoms, or personal/family history of hematologic disorders. She was taking a combined oral contraceptive (levonorgestrel 150 mcg, ethinylestradiol 30 mcg), was a non-smoker, and had previously competed in swimming.

**Fig. 1:**
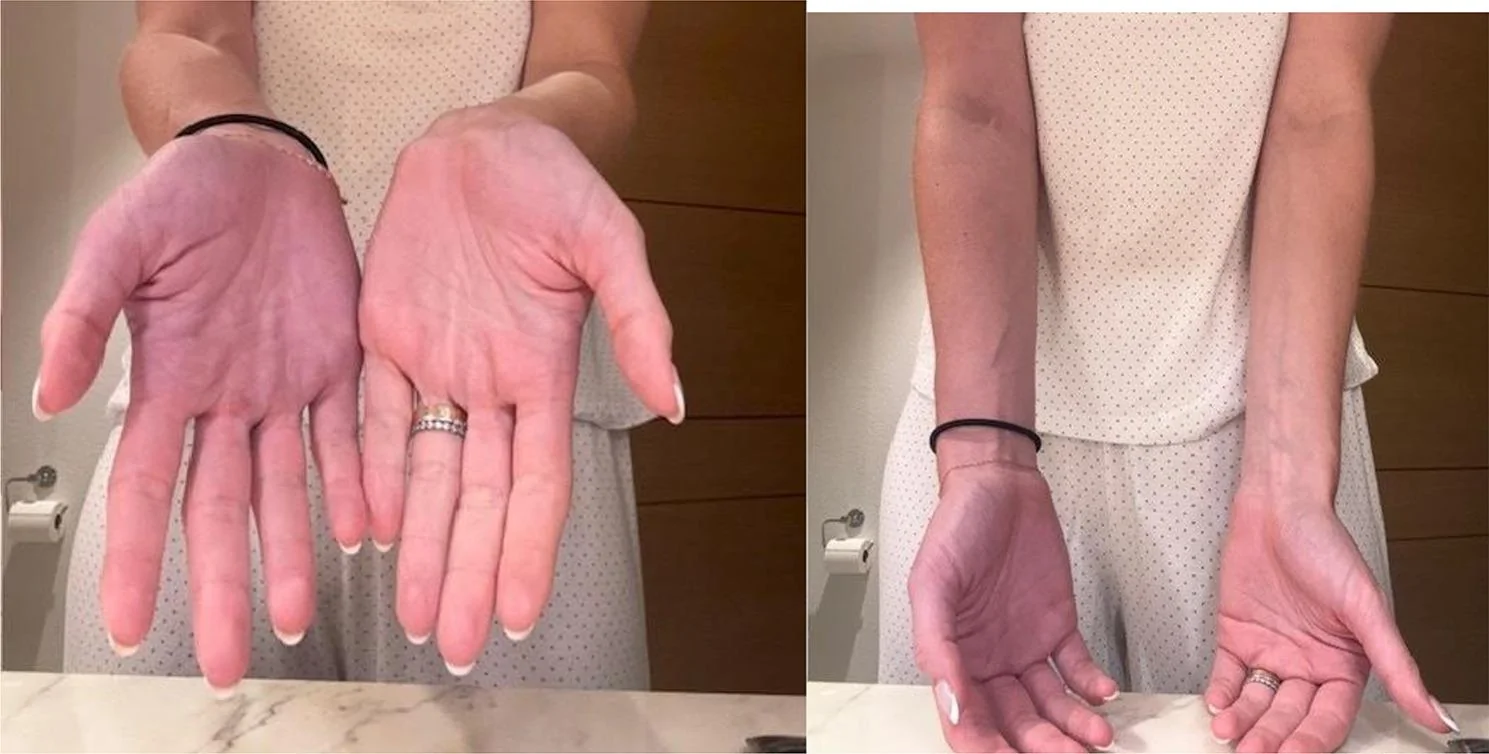
Cyanosis and swelling of the right upper extremity. Photos, taken a couple of weeks earlier the ER visit

Physical examination revealed slight swelling of right arm without pain or cyanosis. A < 1 cm non-mobile medial subaxillary mass was palpable, reproducing tenderness. Neurovascular examination was normal, with symmetric radial pulses and capillary refill < 3 s. Cervical spine and shoulder examinations were unremarkable except for mild right suprascapular muscle tenderness. The patient provided photographs taken two weeks earlier, showing cyanosis and swelling of the right upper extremity (Fig. 1).

**Fig. 2:**
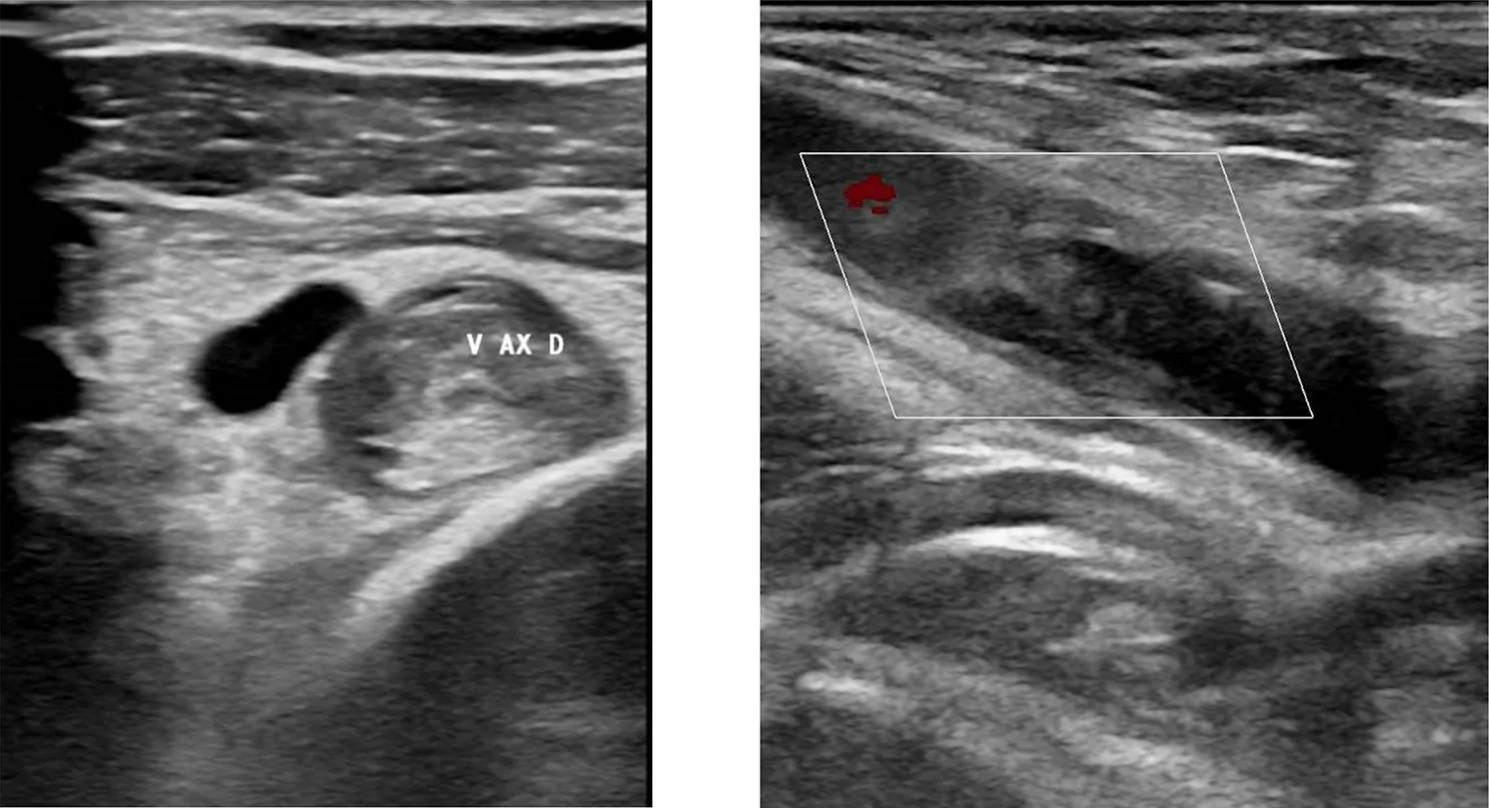
Axial and longitudinal ultrasound views of a thrombus in the right axillary and subclavian veins

Shoulder radiographs showed no fractures or bone disease. Laboratory tests revealed normal hemoglobin and platelets, elevated D-dimer (900 ng/mL, cut-off value 500ng/ml), and mildly increased C-reactive protein (CRP) (14 mg/L, normal value < 5 mg/) without leukocytosis. A complete venous ultrasound confirmed acute occlusive deep vein thrombosis of the distal and middle axillary and subclavian veins (Fig. 2).

**Fig. 3:**
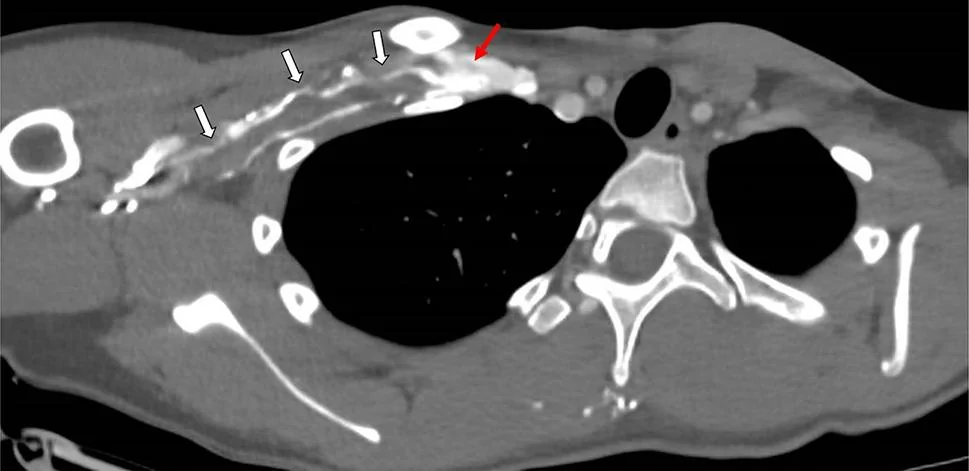
Upper thoracic phlebo-CT demonstrating right axillary thrombosis (white arrows), associated with stenosis on raising the arm of the axillary-subclavian (red arrow)

Rivaroxaban was initiated at a curative dose (15 mg twice daily for three weeks, then 20 mg once daily for three months). Thoracic CT phlebography, with provocative maneuvers, confirmed right axillary thrombosis extending into the thoracic outlet with dynamic stenosis on arm elevation at the axillary–subclavian junction, consistent with thoracic outlet syndrome. Similar findings were documented contralaterally, without thrombosis (Fig. 3).

**Fig. 4:**
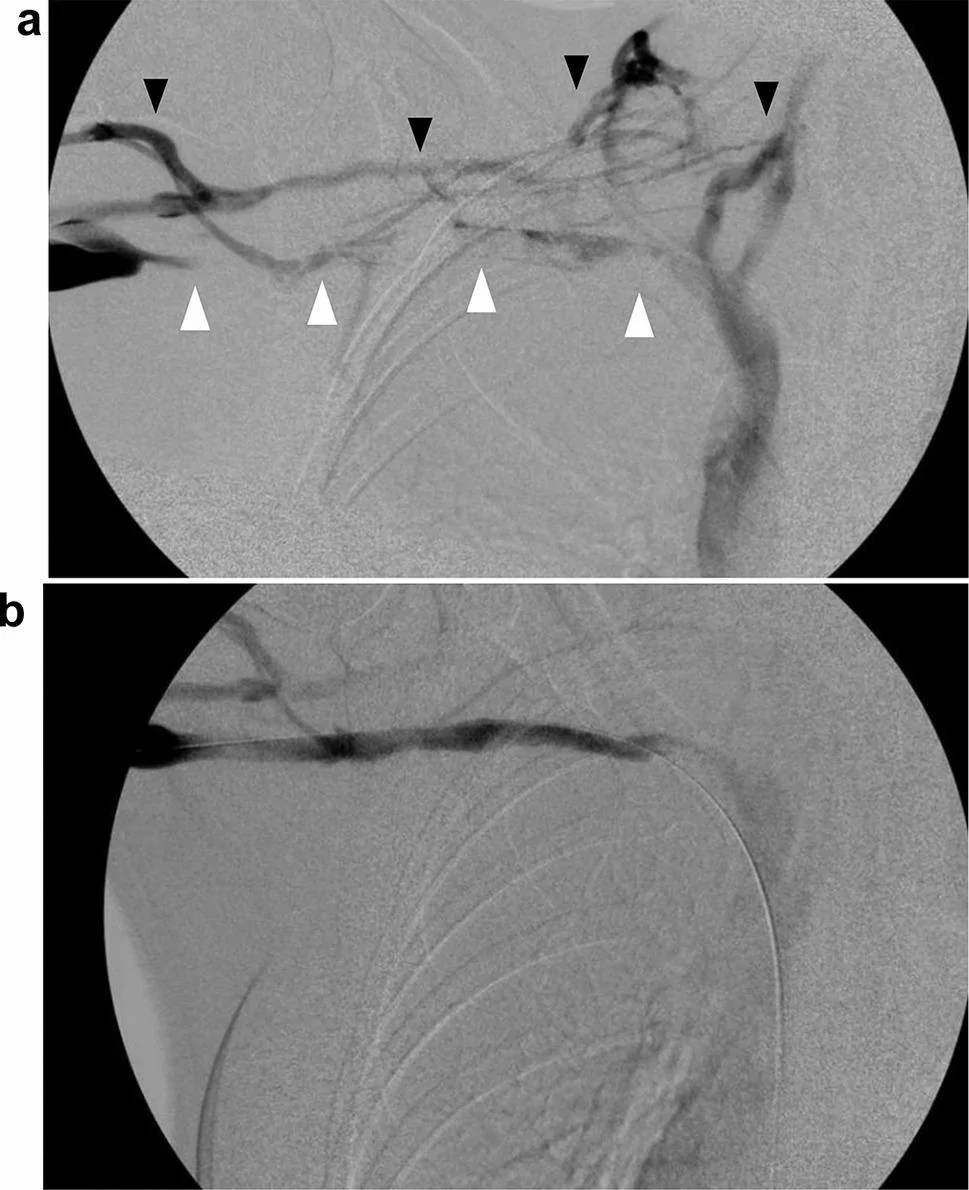
**A**) phlebography of the occluded axillary-subclavian veins (white arrow heads), and collaterals (Black arrow heads) **B**) Phlebography after thrombectomy with minimal collateral flow and residual stenosis at the thoracic inlet

Eighteen days later, because of persistence of symptoms, thrombus burden unchanged under anticoagulation, percutaneous mechanical thrombectomy and venoplasty were performed. Post-procedural phlebography showed improved venous drainage with abolition of collateral flow, and intravascular ultrasound (IVUS) showed persistence of a 70–80% residual stenosis (Fig. 4). Dynamic testing was performed during IVUS examination.

At seven days, a venous ultrasound showed the patency of the axillary-subclavian veins with persistent proximal stenosis, but with full symptoms resolution. Surgical first-rib resection and veinoplasty were planned to prevent recurrence.

## Discussion

This case illustrates the diagnostic path of PSS secondary to vTOS, highlighting the diagnostic challenges in the ED evaluation and the importance of considering patient-provided information. PSS is a primary form of upper extremity deep vein thrombosis (DVT), accounting for 10–20% of cases, with an incidence of 0.5–1 per 100,000 annually [1]. It is strongly associated with venous thoracic outlet syndrome (vTOS) and sports activities, with repetitive subclavian–axillary vein compression leading to endothelial injury, fibrosis, venous stenosis, and thrombosis [1–3]. The mechanisms often involve anatomical or functional factors such as variants in the insertion of the costoclavicular ligament, scalene muscle hypertrophy, or, less commonly, cervical ribs or fibrous bands from C7 [2, 3]. While oral contraceptive use may contribute to hypercoagulability, its role is considered less significant [2, 3]. PSS typically affects young, physically active individuals engaged in repetitive upper limb movement for sport or work [2–4]. In this regard, it should be noted that our patient had been a competitive swimmer.

Typical symptoms of PSS include persistent arm swelling and cyanosis [2], sometimes with prominent superficial veins in the neck, chest, or arm; known as Urschel’s sign [5–7]. Although generally more severe and constant than in intermittent exertional vTOS, symptoms may be intermittent or absent at rest, as in our patient. Diagnosing PSS in the ED is challenging as the presentation may be subtle, nonspecific, or asymptomatic [8]. Transient swelling or discomfort can lead to underestimation of severity, and some patients present only with exertional pain, without clear thrombosis signs [8]. Urschel’s sign is inconsistent [7–13], most cases involve the dominant arm, and absence of classical risk factors—such as repetitive upper limb effort—does not exclude PSS [7, 9, 11]. Neurological or arterial thoracic outlet syndrom, upper limb or thoracic tumors—including Pancoast tumors—and various neuropathies can mimic PSS, leading to misdiagnosis if not considered early [14].

Concerning diagnosis, the combined use of a clinical prediction score (Costans score) and d-dimer avoid the need of imaging in about 20% of patients [2]. About imaging, point-of-care ultrasound (POCUS) Doppler can be the first-line tool for rapid, non-invasive detection of upper extremity deep vein thrombosis in the ED [15, 16]. However, if POCUS is inconclusive, guidelines recommend a complete venous US also useful to identify a differential diagnosis [17, 18]. Contrast CT venography should be limited to selected cases to better assess anatomy and venography remaining the gold standard when treatment is indicated [2, 12]. In recent years, the use of intravascular ultrasound (IVUS) has become widespread [19]. IVUS is an imaging technique in which ultrasound evaluation of the vascular structures is accomplished by passing an ultrasound probe through the vessel lumen. This allows precise and dynamic vessel diameter assessment and cross-sectional area reduction. Two recent papers suggested that in patients with PSS, IVUS would be able to detect greater levels of stenosis than venography alone [20, 21].

As for any venous thromboembolic episode, anticoagulation remains the cornerstone of treatment. However, anticoagulation alone leaves up to 46% of patients with persistent symptoms [22]. Early management combining catheter-directed thrombolysis (CDT) with first-rib resection (FRR) achieves durable symptom resolution in up to 96% [22]. Two meta-analyses recommend this combined approach as first-line [22, 23]. 3D printing has become an important tool in modern medicine. In the context of upper limb cyanosis, the actual role of 3D printing remains indirect and largely adjunctive. It could assist in visualizing complex vascular anatomy, planning surgical or interventional procedures, or developing customized supportive devices [24]. Anticoagulation therapy should be continued for at least 3 months. However, in cases of persistent risk factors (e.g., patients with residual stenosis and persistent compression of the vein), long-term anticoagulation therapy at a reduced dose should be considered in order to prevent recurrence.

PSS management requires multidisciplinary collaboration for rapid diagnosis, anticoagulation, recanalization and definitive decompression [2, 16]. Criteria for early intervention are severity of symptoms. In this case the patient was mildly symptomatic and refused intervention at first. In case of delayed diagnosis, complications may occur in up to 26.7% of cases — most often pulmonary embolism [4] and, without treatment, may include post-thrombotic syndrome, recurrent thrombosis, or functional impairment [25]. Pulmonary embolism develops in 7–20% of cases [7], and a patent foramen ovale increases the risk of paradoxical embolism and stroke [26]. Early diagnosis and surgery are key to prevention [25]. Emergency physicians should maintain high suspicion for unexplained upper limb symptoms, even without classic risk factors [13].

## Conclusion

This case demonstrates that PSS can present with mild or intermittent symptoms. It highlights the value of careful history-taking, including review of patient-provided photographs, in revealing transient events not captured during examination. In the ED, early vascular imaging should be considered for unexplained, recurrent upper limb discoloration or swelling, allowing timely treatment and prevention of severe complication.

## Data Availability

All data generated or analysed during this study are included in this published article.

## Abbreviations

PSS: Paget–Schroetter syndrome
ED: Emergency department
VTOS: Venous Thoracis outlet syndrom
CT: Computerized tomography scan
CRP: C Reactive protein
POCUS: Point-of-care ultrasound
CDT: Catheter-directed thrombolysis
FRR: First-rib resection
DVT: Deep vein thrombosis
