# Correction: Intermittent upper limb cyanosis revealing Paget–Schroetter syndrome: the value of patient-provided photographs

**DOI:** 10.1186/s12245-026-01384-x

**Published:** 2026-09-14

**Authors:** Marine Desjardins, Giulia Casutt, Marco Fresa, Jean Perentes, Adriano Alatri, Pierre-Nicolas Carron

**Affiliations:** 1 Emergency Department, Lausanne University Hospital, Rue du Bugnon 44, Lausanne, CH-1011 Switzerland; 2 Division of Angiology, Lausanne University Hospital, Chemin du Mont-Paisible 18, Lausanne, CH-1011 Switzerland; 3 Division of Thoracic Surgery, Lausanne University Hospital, Rue du Bugnon 46, Lausanne, CH-1011 Switzerland; 4 Emergency Department, Lausanne University Hospital and University of Lausanne, Rue du Bugnon 44, Lausanne, CH-1011 Switzerland

**Correction: International Journal of Emergency Medicine (2026) 19:174**

**https://doi.org/10.1186/s12245-026-01282-2**

Following publication of this article, the authors reported that all given and family names had been transposed. 

The names provided in this Correction are accurate.

The original article has been corrected.

